# The Contribution of Ageing to Hospitalisation Days in Hong Kong: A Decomposition Analysis

**DOI:** 10.15171/ijhpm.2016.108

**Published:** 2016-08-17

**Authors:** Chi Leung Kwok, Carmen KM Lee, William TL Lo, Paul SF Yip

**Affiliations:** ^1^HKJC Centre for Suicide Research and Prevention, The University of Hong Kong, Pokfulam, Hong Kong.; ^2^Department of Social Work and Social Administration, The University of Hong Kong, Pokfulam, Hong Kong.; ^3^Kwai Chung Hospital, Hospital Authority, Kwai Chung, Hong Kong.

**Keywords:** Decomposition, Hospitalisation Days, Ageing, Length of Stay (LOS), Patient Discharge, Hong Kong

## Abstract

**Background:** Ageing has become a serious challenge in Hong Kong and globally. It has serious implications for health expenditure, which accounts for nearly 20% of overall government expenditure. Here we assess the contribution of ageing and related factors to hospitalisation days in Hong Kong. We used hospital discharge data from all publicly funded hospitals in Hong Kong between 2001 and 2012.

**Methods:** A decomposition method was used to examine the factors that account for the change of total hospitalisation days during the two periods, 2001-2004 and 2004-2012. The five factors include two demographic factors – population size and age-gender composition – and three service components – hospital discharge rate, number of discharge episodes per patient, and average length of stay (LOS) – which are all measured at age-gender group level. In order to assess the health cost burden in the future, we also project the total hospitalisation days up to 2041, for a range of scenarios.

**Results:** During the decreasing period of hospitalisation days (2001-2004), the reduction of LOS contributed to about 60% of the reduction. For the period of increase (2004-2012), ageing is associated with an increase in total hospitalisation days of 1.03 million, followed by an increase in hospital discharge rates (0.67 million), an increase in the number of discharge episodes per patient (0.62 million), and population growth (0.43 million). The reduction of LOS has greatly offset these increases (-2.19 million days), and has become one of the most significant factors in containing the increasing number of hospitalisation days. Projected increases in total hospitalisation days under different scenarios have highlighted that the contribution of ageing will become even more prominent after 2022.

**Conclusion:** Hong Kong is facing increasing healthcare burden caused by the rapid increase in demand for inpatient services due to ageing. Better management of inpatient services with the aim of increasing efficiency and reducing LOS, avoidable hospitalisation and readmission, without compromising patient satisfaction and quality of service, are crucial for containing the rapid and enormous increases in total hospitalisation days for Hong Kong. The results would be relevant to many rapidly ageing societies in this region.

## Background

A rapidly ageing demographic is a growing problem for many high- and middle-income countries. The impact of an ageing population on healthcare expenditures has been widely discussed over the past three decades.^1-12^ While some studies have suggested that rapid growth in the older adult population (defined as ages 65 and above) is a significant cost-driver for healthcare services, through mechanisms such as increased utilisation due to longer lifespan,^1-3,6,8,10^ some have argued that the ultimate effect on healthcare expenditure is mild.^5,7,9,11,12^ In either case, the magnitude and rate of the increases in the demand for healthcare services relating to age-associated medical conditions has left many countries unprepared.^13-18^ This has placed a significant financial burden on many governments. In Hong Kong, medical and health service expenditures have been increasing and now account for 16.8% of recurring government expenditures.^19^ This figure is high in comparison to most countries and ranks in the top 30% worldwide in terms of public expenditure per capita.^20,21^ It has been projected that total health expenditures in the public sector will increase from 2.9% of Hong Kong’s gross domestic product (GDP) in 2004 to 5.5% in 2033.^22^ Various measures have been proposed to contain the cost of healthcare, including improvements in the surveillance and monitoring of morbidity, especially among older adults; community care; co-payment for treatment; well-organised coordination between healthcare and social services; health and wellness promotion; and disease prevention programmes within the community. Provision of suitable medical and health services is a major public health measure that will improve the overall well-being of the community.^23^

Hospitalisation is one of the major concerns and the major contributor to a country’s healthcare costs. Also, older patients are generally associated with higher severity of illness, greater complexity and greater comorbidity, leading to longer stays in hospitals and higher hospital costs.^1,3,10,15^ It is usually the greatest among all healthcare expenditures.^24,25^ In Hong Kong, cost of inpatient services represents 55% of healthcare expenditures in public hospitals managed by the Hospital Authority (HA),^26^ a government subsidised and independent body responsible for the oversight of all public hospitals/institutions and outpatient clinics in Hong Kong. Public funding from the government budget supports almost all recurrent expenditures of the public healthcare sector, while only 5% are charged to users. The budget allocation from government to hospitals is a complicated yet simple process. A lump sum is given to HA each year, and HA in turn allocates budgets to individual hospitals based on their baseline activities, case-mix, and population served. Outside this, there are 11 registered private hospitals in Hong Kong to date. They are generally not subsidised and all services are charged to patients or private health insurance. In recent years, due to the increasing demand for medical and health services from the community, public funding has also started supporting one-off capital works for new private hospitals, and designating public-private partnership programmes, like cataract surgery and colonoscopy screening.

A previous study used a decomposition method, based on the ratio of hospitalisation days at two time points, to examine various factors in order to explain the changes in hospitalisation in Hong Kong.^27^ However, the ratio formulation in that paper could not incorporate the demographic factors in terms of population size and age-gender composition, and therefore, was not able to detect how these changes affected hospitalisation days. The contribution of ageing, which is believed to be one of the most important factors in increasing utilisation of inpatient service, has yet to be determined. It is important to incorporate the past decade’s changes in population distribution in understanding changes in hospitalisation, especially when analysing rapidly ageing societies such as Hong Kong. Also, other neighbouring high-income countries in the region, such as South Korea, Japan, and Taiwan are undergoing this ageing process rapidly, due to low fertility rates and lengthening life expectancy. For example, Hong Kong’s older adult population doubled from 5.5% to 11.1% between 1976 and 2001^28^ — it took only 25 years for Hong Kong to double their proportion of older adult population, whereas it took France and other Western countries more than 100 years. Furthermore, if current demographic patterns continue, it is predicted that by 2029 26% of Hong Kong’s population will be older adults, and by 2041, 30%.^29^ It is crucial to have some empirical assessment of the contribution of ageing to hospitalisation days, for effective healthcare planning.

Some decomposition methods, making use of the difference between two total numbers, have been previously used to decompose increase in number of death^30^ and costs of hosptailisations^10^ to measure the contribution of change in demographic factors. In this study, we adopted another decomposition analysis, namely structural decomposition,^31^ to examine the change in total hospitalisation days. By doing so, we were able to assess the relative contribution of population age-gender composition and population size, as well as other related factors, including hospital discharge rate, number of discharge episodes per patient in a year (which partly reflects readmission) and average length of stay (LOS) in hospital. We examined the factors contributing to the changes in total hospitalisation days during the decreasing period (2001-2004) and the increasing period (2004-2012). In order to assess the sustainability of our present system, we also projected the total hospitalisation days for the next three decades, up to 2041, under different scenarios. During the study period 2001-2012, Hong Kong’s population increased from 6.71 million in 2001 to 6.78 in 2004, and then to 7.15 million in 2012,^32^ an average annual growth of 0.6%. The corresponding growth rate in older adults was 2.4%, from 11.2% of the total population to 12.1% and then to 13.7%. The population size is projected to increase to 8.22 million in 2041, with 30.3% of the population consisting of older adults aged 65 or over. Among these, over 60% will be aged 75 and above.^29^

## Methods

### Data

To illustrate the factors contributing to the change in hospitalisation days in Hong Kong, population data and data on hospital discharges were collected from the Census and Statistics Department (C&SD) and HA, respectively. These data are readily available from the website of C&SD and HA. The census statistics for Hong Kong’s resident population are stratified by gender and five-year age group, with an open age group for those aged 85 and above. The HA’s annual statistics reports provided necessary and sufficient aggregated inpatient data for the current decomposition analysis, including the number of inpatients, number of discharge episodes (including death), and total hospitalisation days by gender, age group (0-4, 5-14, 15-44, 45-64, 65-74, and 75 and over), and year. The aggregated inpatient data included records from all 41 public hospitals in Hong Kong, which provided 27.2 thousand hospital beds as of 2012-2013,^26^ and covered almost 80% of inpatient services,^33^ including acute, convalescence, infirmary, mentally ill and mentally handicapped services. Data from private hospitals are not available and they were not included in the analysis. In terms of services, private hospitals provide a range of clinical services similar to public ones and treat all types of patients except psychiatric inpatients. However, due to the difference in funding, services provided by private hospitals are much more expensive. Therefore, patients using private hospitals are generally wealthier and have higher socio-economic status.

All statistics shown in this paper have excluded records with unknown gender or age, which accounted for less than 0.01% of total hospitalisation days each year.

### Notation

*i* – Age-gender group (gender: male and female; age group: 0-4, 5-14, 15-44, 45-64, 65-74, and 75+; 12 groups in total)

*D_t_* – Total hospitalisation days at year t

*D_it_* – Total hospitalisation days in group i at year t *E_it_* – Number of hospital discharge (and death) episodes in group i at year t

*I_it_* – Number of distinct hospitalised individuals in group i at year t

*P_it_* – Population size in group i at year t

*P_t_* – Total population size at year t

### Mathematical Formulation

Examining the difference in total hospitalisation days between the time points can provide a direct understanding of the changes of the financial burden on the healthcare system, which is more straightforward than a decomposition of its mean. Decomposition analysis in demography generally deals with the difference between two rates/means. However, we saw that the mathematical expression would be no different from normal if considering the difference of two total numbers.

Total hospitalisation days at year *t* can be expressed as the product of five components summed across age-gender group, ie:

(1) $$D_{t}=\sum_{i}^{}D_{it}=\sum_{i}^{}\frac{D_{it}}{E_{it}}\times \frac{E_{it}}{I_{it}}\times \frac{I_{it}}{P_{it}}\times \frac{P_{it}}{P_{t}}\times P_{t}=\sum_{i}^{}l_{it}\times \mu_{it}\times r_{it}\times \pi_{it}\times P_{t}$$

The five components in Equation (1) are: (*i*)* l_it_=D_it_/E_it_* , LOS in each age-gender group *i* at year *t*; (*ii*) *μ_it_= E_it_/I_it_* , the annual average number of discharge episodes per patient; (*iii*) *r_it_= I_it_/P_it_*, the age-gender-specific hospital discharge rate; (*iv*)* π_it_ = P_it_/P_t_*, the proportion of population in group *i* to the total population, and (*v*) *P_t_ = ∑_i_P_it_*, the total population size.

Structural decomposition analysis,^31^ which was developed in the field of economics, was thought to be suitable for this study. To measure the change of total hospitalisation days between two years, the decomposition formulation of the difference takes the form:

(2) $$\Delta D=\underset{\text{i}}{\sum }\Delta l_{i}\times \mu_{i2}r_{i2}\pi_{i2}P_{2}+\underset{\text{i}}{\sum }l_{i1}\times \Delta \mu_{i}\times r_{i2}\pi_{i2}P_{2}+\underset{\text{i}}{\sum }l_{i1}\mu_{i1}\times \Delta r_{i}\times \pi_{i2}P_{2}+\underset{\text{i}}{\sum }l_{i1}\mu_{i1}r_{i1}\times \Delta \pi_{i}\times P_{2}+\underset{\text{i}}{\sum }l_{i1}\mu_{i1}r_{i1}\pi_{i1}\times \Delta P$$

The first term gives the magnitude of change in total hospitalisation days contributed to by the change in LOS, weighted by *μ*_i2_*r*_i2_*π*_i2_*P*_2_. The second term accounts for the contribution due to the change in the number of discharge episodes per patient, weighted by *l*_i1_*r*_i2_*π*_i2_*P*_2_, and so on. The idea behind the decomposition is to shift the weight from the second time point to the first time point, one by one, when moving to the next factor. The formulation is simple and exact. However, this decomposition expression is not unique and depends on the order of factors. In total, there are 120 equivalent expressions. As the number of equivalent decomposition expressions increases with the increasing number of factors, Dietzenbacher and Los^31^ suggested giving the contribution of factors by the average of two polar decompositions, which would give results close to the arithmetic mean of all decompositions. In this paper, we adopt the latter approach to find the exact mean of all combinations. The method used to decompose the increase in total hospitalisation costs in a recent study^10^ was in fact equivalent to one of the many different expressions formulated by structural decomposition analysis.

It should be noted that *P*_t_ denotes the total population size at year *t*, and therefore, there is no subscript *i* in the equation. Decomposition results from the last term were not appropriate to be presented by the age-gender group and will be presented as a lump sum.

### Projection

To assess the contribution of future ageing to hospitalisation, total hospitalisation days were projected up to 2041. We first assumed that the hospital discharge rate, number of discharge episodes per patient and LOS were maintained at 2012 age-gender group levels for the next three decades, up to 2041. Decomposition analysis was then applied to decompose the increase of total hospitalisation days between 2012 and each targeting year into two demographic factors: change in population structure and population growth. This serves as a base scenario. After that, to reflect possible future changes in policy, social context and market for hospital care, such as shifts of services from hospital settings to the ambulatory sectors, effective disease prevention programmes, and changes in disease spectrum, scenario analyses were performed by applying different values in the improvement of the three service components. In particular, in each scenario, we assumed a 5%, 10%, or 15% decrease of different service components from 2012-2041 at age-gender group level, and applied these values to calculate the total hospitalisation days in 2041. Decomposition analysis was then applied to decompose the difference between 2012 and 2041 to examine the contribution of each component.

## Results

**Figure 1:**
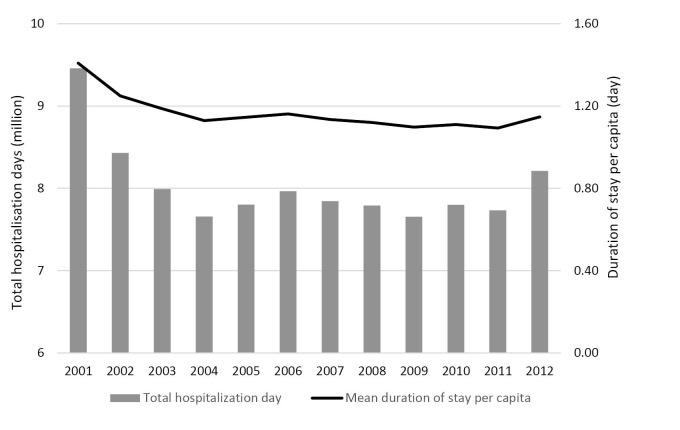
Total Hospitalisation Days of Publicly Funded Hospitals and Duration of Stay Per Capita in Hong Kong, 2001-2012.

During the period 2001 to 2012, the number of inpatients discharged from public hospitals increased steadily by 1.48% per annum, from 607 000 to 714 000. An exception was found in 2003, in which a large reduction was recorded. This was due to the outbreak of severe acute respiratory syndrome (SARS), when many of the hospital services for the community were disrupted. People avoided going to hospital for fear of contracting SARS in the hospital during the outbreak in the first half of 2003. However, we note that the number of hospital beds in HA hospitals decreased by 7.97% between 2002 and 2012.^34^ As reported by HA, the total hospitalisation days of inpatient discharges and deaths in public hospitals decreased greatly, from 9.46 million in 2001 to 7.66 million in 2004 (Figure 1). It remained at a steady level at around 7.80 million between 2005 and 2011, and then rebounded to 8.21 million in 2012. Therefore, the decomposition analysis in this paper was separated into two periods – the decreasing period 2001-2004 and increasing period 2004-2012 – in order to investigate the decreasing trends during the early period as well as the recent increases for 2011-2012. Meanwhile, duration of hospital stay per capita also decreased, by 19.85%, from 1.41 days in 2001 to 1.13 days in 2004, and then remained at a similar level until 2012 (1.09-1.15 days).

Every year, 31.38%-35.18% of inpatients, comprising 38.20%-42.00% of discharge episodes and 45.31%-50.52% of total hospitalisation days, were older adults, which is over-representative of the population; as of 2012, older adults only made up 13.70% of the population. The majority of the increase came from those aged 75 or older, which increased by 63.78% throughout the study period, whereas the number of inpatients between the ages of 65 and 74 reduced slightly, by 5.55%. The number of inpatients between the ages of 45 and 64 also increased, by 42.80%.

Although the number of inpatients showed growth, LOS decreased by 11.51% from 7.82 to 6.92 days between 2001 and 2004, and then decreased another 23.51% to 5.29 days in 2012 (in total there was a 32.31% decrease for the period 2001-2012). However, we found that the decrease in LOS was not homogenous across genders and age groups, and was reflected most significantly in males between the ages of 45 and 64 (from 9.77 days to 5.14 days for the period 2001-2012). In addition, the number of discharge episodes per patient increased between 2001 and 2012 in all age-gender groups, except for female children and adolescents between the ages of 5 and 14. The largest increase was found among inpatients aged 45-74. Change in total hospitalisation days due to decrease in LOS was partially offset by the increased number of episodes per patient. The crude discharge rate increased by 10.33% from 90.40 to 99.74 per 1000 population between 2001 and 2012. The gender-specific discharge rate for females was 1.04 to 1.14 times that for males, but this difference has narrowed in recent years. Despite the large increase in the number of middle-aged (45-64) and elderly (75 or older) inpatients, the corresponding discharge rates decreased or remained constant. This means the large increase in the number of patients in the past decade was mainly due to a shift in the population structure (ie, ageing). Detailed breakdown of the figures by age group and gender for three selected years (2001, 2004, and 2012) can be found in Table 1.

**Table 1:** Summary of Population and Discharges From Publicly Funded Hospitals in Hong Kong, by Selected Year 2001, 2004, and 2012

**Age Group**	**Gender**	**LOS**	**No. of Discharges Per Patient**	**Discharge Rate Per 1000 Population**	**Population Structure (%)**	**Total Population Size (000)**
2001						
0-4	F	4.20	1.44	192.46	1.95	
	M	3.98	1.56	231.52	2.11	
5-14	F	4.20	1.60	35.41	5.95	
	M	3.93	1.52	50.84	6.35	
15-44	F	4.49	1.77	71.76	26.56	
	M	12.55	1.65	42.92	23.81	
45-64	F	6.20	1.96	85.93	10.62	
	M	9.77	2.23	84.41	11.42	
65-74	F	8.06	2.14	178.56	3.42	
	M	9.02	2.48	206.82	3.43	
75+	F	10.15	2.40	341.73	2.62	
	M	9.36	2.67	355.64	1.75	
Overall		7.82	1.99	90.40	100.00	6714.30
2004						
0-4	F	3.82	1.38	262.39	1.61	
	M	3.82	1.49	287.19	1.74	
5-14	F	5.16	1.54	26.75	5.57	
	M	3.99	1.52	39.62	5.89	
15-44	F	4.88	1.65	61.80	25.82	
	M	9.46	1.67	35.39	22.32	
45-64	F	5.74	2.00	67.58	12.34	
	M	7.38	2.24	73.76	12.64	
65-74	F	7.13	2.20	145.93	3.48	
	M	6.91	2.42	189.52	3.53	
75+	F	9.43	2.33	307.74	3.02	
	M	8.55	2.56	343.18	2.03	
Overall		6.92	1.97	82.64	100.00	6783.50
2012						
0-4	F	3.33	1.51	300.42	1.76	
	M	3.24	1.59	327.82	1.91	
5-14	F	3.22	1.54	38.73	3.73	
	M	3.01	1.56	52.68	3.97	
15-44	F	4.29	1.80	70.36	24.33	
	M	7.10	1.77	38.50	19.17	
45-64	F	4.63	2.29	77.26	16.35	
	M	5.14	2.53	83.03	15.09	
65-74	F	4.96	2.42	153.31	3.33	
	M	5.10	2.67	190.99	3.46	
75+	F	7.05	2.42	321.44	4.00	
	M	6.44	2.68	358.16	2.91	
Overall		5.29	2.17	99.74	100.00	7154.60

Abbreviations: F, Female; M, Male; LOS, length of stay.

### The Decomposition Results

Table 2 gives the difference of total hospitalisation days for 2001-2004 and 2004-2012 contributed to by the five components. The total of each component was annualised for easier comparison between the two periods of different length. During the decreasing period, total hospitalisation days decreased by 599 718 days per annum on average, representing an annual reduction rate of 6.79%. Decomposition analysis showed that the decrease in LOS, the change in the number of discharges per patient, and the decreased discharge rate in almost all age-gender groups contributed to 63.86%, 8.14%, and 57.88% of the reduction, respectively. By contrast, growth in population size and the change in population structure would contribute an increase to total hospitalisation days of 29 254 and 149 933 days per annum, respectively, representing a 0.31% and 1.56% annual increase from 2001 (out of 9.46 million). During the increasing period, total hospitalisation days increased by an average of 69 062 days (+0.87%) per annum. Decomposition analysis showed extraordinary values for each component, indicating that the underlying pattern was greatly altered. Decreases in LOS (-273 989 days per annum) solely offset the positive contributions of the other four factors, but at a lower rate than before (-382 980 days). Change in population structure alone contributed to a 128 685 increase, of which 81.53% was contributed by individuals aged 75 or above. The two demographic components together consistently contributed to an increase of around 180 000 days per annum. The contribution of children and adolescents to the change in total hospitalisation days was small.

**Table 2:** Decomposition Results of Total Hospitalisation Days, 2001-2004 and 2004-2012 (% of Total)

**Age group**	**Gender**	**LOS**	**No. of Discharges** **Per Patient**	**Discharge Rate**	**Population Structure**	**Population Growth**	**Total**
2001-2004						
0-4	F	-14 680 (0.82)	-6477 (0.36)	47 654 (-2.65)	-29 040 (1.61)		-2543 (0.14)
	M	-8331 (0.46)	-10 036 (0.56)	43 186 (-2.40)	-38 579 (2.14)		-13 760 (0.76)
5-14	F	18 309 (-1.02)	-3318 (0.18)	-24 703 (1.37)	-5885 (0.33)		-15 598 (0.87)
	M	1912 (-0.11)	-214 (0.01)	-27 916 (1.55)	-8313 (0.46)		-34 530 (1.92)
15-44	F	79 232 (-4.40)	-66 503 (3.70)	-140 993 (7.84)	-26 866 (1.49)		-155 129 (8.62)
	M	-311 598 (17.32)	13 709 (-0.76)	-213 937 (11.89)	-72 305 (4.02)		-584 131 (32.47)
45-64	F	-53 809 (2.99)	15 394 (-0.86)	-167 745 (9.32)	105 039 (-5.84)		-101 121 (5.62)
	M	-341 973 (19.01)	5670 (-0.32)	-165 430 (9.19)	124 251 (-6.91)		-377 481 (20.98)
65-74	F	-76 888 (4.27)	17 302 (-0.96)	-125 462 (6.97)	11 287 (-0.63)		-173 762 (9.66)
	M	-241 040 (13.40)	-23 712 (1.32)	-79 319 (4.41)	26 320 (-1.46)		-317 751 (17.66)
75+	F	-105 515 (5.86)	-44 653 (2.48)	-149 514 (8.31)	206 526 (-11.48)		-93 156 (5.18)
	M	-94 559 (5.26)	-43 529 (2.42)	-37 226 (2.07)	157 363 (-8.75)		-17 951 (1.00)
Total		-1 148 941 (63.86)	-146 367 (8.14)	-1 041 405 (57.88)	449 798 (-25.00)	88 761 (-4.88)	-1 799 153 (100.00)
Annualised total		-382 980	-48 789	-347 135	149 933	29 254	-599 718
2004-2012						
0-4	F	-23 544 (-4.26)	14 526 (2.63)	23 038 (4.17)	15 126 (2.74)		29 146 (5.28)
	M	-34 981 (-6.33)	14 571 (2.64)	28 048 (5.08)	18 900 (3.42)		26 538 (4.80)
5-14	F	-31 253 (-5.66)	-237 (-0.04)	25 320 (4.58)	-26 679 (-4.83)		-32 850 (-5.95)
	M	-23 663 (-4.28)	1995 (0.36)	24 293 (4.40)	-33 116 (-5.99)		-30 491 (-5.52)
15-44	F	-117 004 (-21.18)	79 397 (14.37)	118 271 (21.41)	-54 126 (-9.80)		26 538 (4.80)
	M	-216 613 (-39.21)	46 609 (8.44)	64 086 (11.60)	-115 206 (-20.85)		-221 124 (-40.02)
45-64	F	-174 067 (-31.51)	107 989 (19.55)	107 136 (19.39)	224 065 (40.55)		265 122 (47.99)
	M	-407 586 (-73.77)	133 254 (24.12)	132 739 (24.03)	198 805 (36.98)		57 212 (10.36)
65-74	F	-177 356 (-32.10)	45 831 (8.30)	24 379 (4.41)	-23 163 (-4.19)		-130 309 (-23.59)
	M	-213 341 (-38.61)	71 394 (12.92)	5458 (0.99)	-14 442 (-2.61)		-150 930 (-27.32)
75+	F	-436 440 (-78.99)	55 337 (10.02)	65 078 (11.78)	418 976 (75.83)		102 951 (18.63)
	M	-336 065 (-60.83)	49 837 (9.02)	50 258 (9.10)	420 340 (76.08)		184 369 (33.37)
Total		-2 191 915 (-396.73)	620 504 (112.31)	668 104 (120.92)	1 029 480 (186.33)	426 324 (77.16)	552 497 (100.00)
Annualised total		-273 989	77 563	83 513	128 685	53 291	69 062

Abbreviations: F, Female; M, Male; LOS, length of stay. Note: Figures may not add up to total due to rounding.

As mentioned, 2012 is the year in which a rapid increase in hospitalisation days was observed (see Figure 1). A sensitivity analysis was, thus, conducted using data from 2004-2011 (details not shown), during which total hospitalisation days rose by 74 630 days only. It consolidated the result that decrease in LOS solely offset the increases contributed by all other factors (-2.20 million in seven years). This result further suggested that the change of LOS from 2011-2012 was so minor (2011: 5.24 days; 2012: 5.29 days) that it could no longer counteract the increased hospitalisation days due to increased hospital discharge rates, change in population structure and population growth, resulting in a large overall increase in one year.

### Projections up to 2041

**Figure 2:**
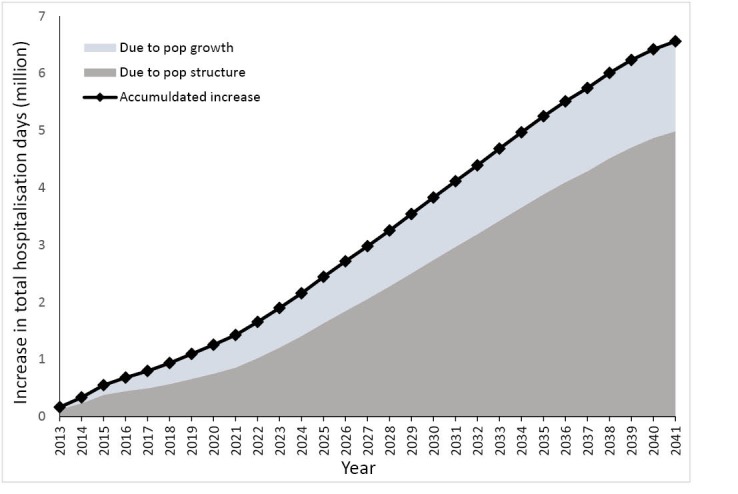
Accumulated Increase in Total Hospitalisation Days (From 2012), Contributed to by Change in Population Structure and Population Growth, up to Year 2041 (Base Scenario).

Figure 2 visualises the projected increase of total hospitalisation days in the future, assuming stable hospital discharge patterns (ie, base scenario). The projected numbers are presented on an accumulating basis from 2012. Over the next few years, total hospitalisation days are expected to increase steadily. However, the contribution of an ageing population will become larger from 2022 onwards. Total hospitalisation days in 2022 are projected to increase by 1 021 592 days contributed to by population ageing, and 631 544 days due to population growth – a total of 20.14% increase from 2012. Between 2012 and 2041, population ageing is predicted to contribute to an increase in total hospitalisation days of 4.99 million (60.77%), and the growth in population size contributes to another 1.57 million (19.10%). In addition, even if the discharge patterns remain unchanged at age-gender group level, the overall hospital discharge rate would still increase by 34.42% due to a large older adult population in 2041. Simply increasing the number of hospital beds is insufficient to cope with expansion at such a rate.

Results from the scenario analysis are summarised in Table 3. Scenario 1 assumed that LOS would further decrease by 5% at every age-gender group in 2041 through service improvement. It would decrease the total projected days by 738 371 and the reduced LOS would contribute to a 568 667 days decrease. The same reduction would be obtained in scenarios 2 and 3, which assumed the same percentage of decrease in the number of discharge episodes per patient and hospital discharge rate, respectively. This was an expected result, as total hospitalisation days was a multiplication of the five components. Scenarios 7 to 9 applied a 10% decrease in each of the three service components to individuals aged 45 and above, who will account for 56% of the total population in 2041. These scenarios reflected future interventions that would mainly target middle-aged group and older adults. Focusing on this 56.26% of the population would bring about 87% of the effect in scenarios 4-6. In all scenarios, change in population age-gender structure would remain a significant factor in driving up total hospitalisation days. We might still restrain this enormous increase by introducing multiple measures that bring large reductions in all three service components (scenario 10).

**Table 3:** Scenario Analysis of Total Hospitalisation Days in 2041 and Decomposition Results From 2012-2041

**Scenario**	**LOS [1]**	**No. of Discharge** **Per Patient [2]**	**Discharge Rate [3]**	**Population Structure**	**Population Growth**	**Total (Different From 2012)**	**Different from base**
Base	-	-	-	4 989 210	1 568 408	6 557 617	-
1 (-5% in [1])	-568 667	-	-	4 861 598	1 526 316	5 819 247	-738 371
2 (-5% in [2])	-	-568 667	-	4 861 598	1 526 316	5 819 247	-738 371
3 (-5% in [3])	-	-	-568 667	4 861 598	1 526 316	5 819 247	-738 371
4 (-10% in [1])	-1 137 335	-	-	4 733 986	1 484 225	5 080 876	-1 476 741
5 (-10% in [2])	-	-1 137 335	-	4 733 986	1 484 225	5 080 876	-1 476 741
6 (-10% in [3])	-	-	-1 137 335	4 733 986	1 484 225	5 080 876	-1 476 741
7 (-10% in [1] for age ≥45)	-930 854	-	-	4 709 021	1 498 010	5 276 178	-1 281 440
8 (-10% in [2] for age ≥45)	-	-930 854	-	4 709 021	1 498 010	5 276 178	-1 281 440
9 (-10% in [3] for age ≥45)	-	-	-930 854	4 709 021	1 498 010	5 276 178	-1 281 440
10 (-15% in [1] [2] [3])	-1 440 163	-1 440 163	-1 440 163	3 952 468	1 227 265	859 243	-5 698 375

Abbreviation: LOS, length of stay.

## Discussion

This paper applies a decomposition analysis to decompose total hospitalisation days by incorporating the contribution made by changes in population. Our results empirically quantified the extent to which the increase during the past decade in hospitalisation days in Hong Kong public hospitals can be attributed to population growth and shifts in population structure. Between 2004 and 2012, population growth and ageing, together with the increased demand for medical and healthcare services, as reflected by the rise in hospital discharge rates (from 82.64 to 99.74 per 1000 population), and number of hospital discharges per patient (from 1.97 to 2.17), have increased the demand of inpatient services by 2.74 million hospitalisation days. Decreases in LOS, from 6.92 to 5.29 days on average, offset this increase. Our previous paper already illustrated the important role LOS plays in determining total hospitalisation days against a 90% overall hospital occupancy and its associated impacts.^27^ This significance was further strengthened in the current study. Decrease in LOS is sometimes treated as an indicator of improvements in efficiency,^35-37^ but efficiency is a complicated matter that depends on a number of factors^38^ and may hardly be represented by a single measure. Thus, it is more important to look into the association between quality of care and LOS. In general, reduction in LOS does not appear to be associated with any deterioration in the quality of care and health outcomes.^37,39,40^ Evidence of such an association in Hong Kong is limited. A local survey conducted in 2010 showed that patient satisfaction with hospitalisation in public hospitals remained high, at 80%.^41^

The establishment of HA has markedly improved the hospital management of Hong Kong. Targeting the increasing medical needs arising from an ageing population, HA has implemented various initiatives emphasising multi-disciplinary participation, and enhanced ambulatory and community care to develop efficient integrated care delivery models since 2000-2001. These have included but are not limited to the establishment of multi-disciplinary rehabilitation ambulatory care centres and nurse-led pre-admission clinics, development of integrated patient care plans from acute to convalescent care, targeting specific disease groups such as stroke, chronic obstructive pulmonary disease and geriatric hip fractures, and expanded community support and clinic services for patients with chronic conditions. These measures initially resulted in huge successes in reducing total hospitalisation days, which were largely contributed to by the decrease in discharge rates and LOS, as shown by our analysis (Table 2: 2001-2004). However, the marginal returns due to further decrease in LOS started diminishing after a few years, being eaten up by the rise in hospitalisation days driven by increases in service demand and public awareness of health conditions. Entering the 2010s, the completion of the overall implementation of the improvement initiatives of hospital management has seen gains plateau off, within the scope of current management strategies and technologies. This has triggered ongoing planning to build hospital services capacity in both the public and private sectors by the Hong Kong government, using renewed management structures to face the challenges of an ageing population in the many years to come.

The pace and magnitude of ageing have posed serious challenges to the carrying capacity of Hong Kong’s public hospital system. While people in Hong Kong are enjoying a longer life span (86.6 for females and 80.9 for males as of 2013),^42^ they are not necessarily healthier.^43^ Due to low total fertility rates, the impact of ageing on Hong Kong’s society is more severe than on most developed countries, through a faster decline in the number of working-age members of the population per retiree. Our results show that, in the past decade, ageing has contributed substantially to changing inpatient demand and will continue to do so. The magnitude of increase contributed to by ageing will become more prominent from 2022 onwards. The increasing demand for inpatient services due to the increasing size of the population of older adults is a predictable, but inevitable, phenomenon. However, our scenario analysis shows that if we can adequately manage the three service components, especially of individuals aged 45 and above, we can still mitigate the pressures that arise from our ageing community. Although the same percentage of decrease in any one of the components would result in the same projected hospitalisation days, they have completely different policy implications, as different strategies and measures are required. In addition, the extent to which these components can further reduce is subject to their variability. Our data in Hong Kong showed that the variability of LOS (coefficient of variation = 0.14) was much higher than the hospital discharge rate (0.07) and number of discharges per patient (0.04).

How much further can LOS be shortened in Hong Kong? Would it affect the quality of health services, treatment outcomes, and healthcare expenditure transfer? There have been numerous interventions aimed at shortening LOS, such as measures taken by Dutch hospitals.^44^ Borghans and colleagues suggested that among 69 Dutch hospitals, if LOS of the 15th percentile hospital (5.7 days) were used as a benchmark, total hospitalisation days could be reduced by 14%.^38^ During the study period, Hong Kong public hospitals recorded a larger decrease in LOS when compared to most the Organization for Economic Co-operation and Development (OECD) countries.^45^ Our performance was similar to the Netherlands (5.2 days in 2012) and was longer than Mexico (3.9 in 2012), Turkey (4.0 in 2012), Norway (4.5 in 2010), Denmark (4.6 in 2010), and the United States (4.8 in 2010). This suggests room for further decreases in LOS, and Hong Kong can benefit from experience of other countries. However, each healthcare system may have a different benchmark. There may exist an optimum length of hospital stay, but it will be affected by medical advancements (eg, outpatient clinics and day ward surgery) as well as local supply (eg, day-care, relevant community service, and public allowance support), and demand factors (eg, morbidity patterns and readmission).^35^ A local evaluation study on the association between LOS and severity of illness may identify opportunities for reducing LOS. More importantly, we should not aimlessly reduce LOS without considering the possible impact on the quality of care and patient satisfaction. Measures for reducing LOS should only be taken if the same level of or even better quality of care and patient satisfaction can be assured. The extent to which further decreases in LOS associate with inpatient demand deserves more attention in Hong Kong. Apart from LOS, multiple initiatives should be developed to target discharge rates and readmissions simultaneously. For example, additional investment in disease prevention programmes, community care, and long-term care programmes to serve patients with chronic health conditions and long-term acute care needs, such as intravenous therapy in nursing homes, have potential to reduce avoidable hospitalisation. Kripalani et al^46^ suggested interventions involving multiple components to reduce readmission rates for patients being discharged to home and post-acute care facilities. It is also important to focus on effective retirement and medical reform that would benefit residents’ health by making use of both public and private healthcare resources, and reduce the burden of healthcare expenditure on the government in the long run.

There are two major limitations to this study. First, we included only discharge data from publicly-funded hospitals in Hong Kong. It is known that public hospitals under HA currently account for about 80% of inpatient services,^33^ but detailed data of inpatients admitted to private institutions in Hong Kong are not available to the authors. Total hospitalisation days contributed by private hospitals are unknown, and therefore this study cannot assess how the change in proportion of population who would use private healthcare services contributes to total hospitalisation days in the public sector. Second, this paper used total hospitalisation days as a proxy for the demand for inpatient services. Obviously, a simple count of this could not fully reflect the actual workload of staff and the cost associated with inpatient services. For uncovering factors that drive up inpatient costs, further research is needed to examine changes in hospital care intensity, such as the number of tests and procedures during a hospitalisation episode.

## Conclusion

Ageing is inevitable and it causes increasing demand for medical and healthcare service in any community. This paper applies a decomposition method to empirically quantify the changes of total hospitalisation days contributed to by five factors, especially due to changes in population composition and size. We believe that this methodology can be applied in different settings to examine the contribution that ageing makes to hospitalisation days, and can therefore, provide evidence for appropriate intervention. In particular, better inpatient management with the purpose of reducing LOS, avoidable hospitalisation and readmission rates are crucial to minimising future increases in total hospitalisation days. The extent of, and measures for, further reduction deserve more attention in Hong Kong public hospitals. It is essential to establish appropriate plans for our healthcare system today, and prepare for the impact that an ageing population will have in the near future. For example, how we enhance community care services and support from family members to reduce LOS and readmissions can be critical elements in managing the continuously increasing inpatient demand, which still represents the greatest healthcare expenditure. The latest innovations in medical technology could also reduce avoidable hospitalisation and LOS after operations. All this information and such improvements are much needed to slow down the increase in hospitalisation days contributed to by rapid ageing.

## Acknowledgements

This work was supported by Strategic Public Policy Research (SPPR) grant on Population Policy at the University of Hong Kong, Pokfulam, Hong Kong (grant number 7003-SPPR-12). The authors would like to thank the anonymous reviewers for their constructive comments that help improve the manuscript.

## Ethical issues

There is no ethical issues as no primary data were collected from any human subjects and only aggregated data was used.

## Competing interests

Authors declare that they have no competing interests.

## Authors’ contributions

CLK, CKML, and PSFY conceived the concept and design of the research. CLK, with the assistance of WTLL, conducted the data collection. CLK and CKML jointly conducted the data analysis, interpreted the result, and also prepared the initial draft of the manuscript. WTLL and PSFY provided critical revision of the manuscript for important intellectual content. PSFY supervised the research and obtained the funding of the project on which the research is based. All authors reviewed and approved the final manuscript.

## Authors’ affiliations

^1^HKJC Centre for Suicide Research and Prevention, The University of Hong Kong, Pokfulam, Hong Kong. ^2^Department of Social Work and Social Administration, The University of Hong Kong, Pokfulam, Hong Kong. ^3^Kwai Chung Hospital, Hospital Authority, Kwai Chung, Hong Kong.

## Key messages

**Implications for policy makers**

- We quantified the changes in hospitalisation days empirically into various components, using a decomposition analysis. Ageing was found to make the strongest contribution to increasing inpatient demand, and will continue to do so.
- The increasing demand for inpatient services due to ageing is a predictable and inevitable phenomenon. Adequately managing length of stay (LOS) in hospital and reducing avoidable hospitalisation and readmission are important, to contain the rate of increasing inpatient demand. Among these, LOS seems to have the largest possibility of further reduction.
- The methodology in this paper can readily be adopted in other settings, to assess the contribution of ageing, so that more focused and targeted measures can be developed.

**Implications for public**

In this paper, we made use of data from all publicly funded hospitals in Hong Kong, which has a rapidly ageing population, to quantify empirically and illustrate how changes in population and hospitalisation patterns have affected the demand for inpatient services, as measured by total hospitalisation days. In particular, ageing and population growth would have tremendously increased total hospitalisation days during the past decade, but this increase was offset by the decrease in length of stay (LOS) per hospital visit. However, the effectiveness of measures to reduce LOS has plateaued and ageing is predicted to become much more significant in increasing inpatient demand in the future. Further reduction in LOS, avoidable hospitalisation, and readmission rates, with no adverse influence on the quality of care and patient satisfaction, is crucial to mitigate the high financial pressures on healthcare arising from an ageing population.
